# Hyperexcitability of Cortical Oscillations in Patients with Somatoform Pain Disorder: A Resting-State EEG Study

**DOI:** 10.1155/2019/2687150

**Published:** 2019-07-09

**Authors:** Qian Ye, Dong Yan, Manlin Yao, Wutao Lou, Weiwei Peng

**Affiliations:** ^1^School of Psychology, Shenzhen University, Shenzhen, China; ^2^Shenzhen Key Laboratory of Affective and Social Cognitive Science, Shenzhen University, Shenzhen, China; ^3^Department of Pain Medicine and Shenzhen Municipal Key Laboratory for Pain Medicine, Shenzhen Nanshan People's Hospital of Shenzhen University Health Science Center, Shenzhen, China; ^4^School of Traditional Chinese Medicine, Southern Medical University, Guangzhou, China; ^5^Department of Medicine and Therapeutics, The Chinese University of Hong Kong, Hong Kong

## Abstract

Patients with somatoform pain disorder (SPD) suffer from somatic pain that cannot be fully explained by specific somatic pathology. While the pain experience requires the integration of sensory and contextual processes, the cortical oscillations have been suggested to play a crucial role in pain processing and integration. The present study is aimed at identifying the abnormalities of spontaneous cortical oscillations among patients with SPD, thus for a better understanding of the ongoing brain states in these patients. Spontaneous electroencephalography data during a resting state with eyes open were recorded from SPD patients and healthy controls, and their cortical oscillations as well as functional connectivity were compared using both electrode-level and source-level analysis. Compared with healthy controls, SPD patients exhibited greater resting-state alpha oscillations (8.5-12.5 Hz) at the parietal region, as reflected by both electrode-level spectral power density and exact low-resolution brain electromagnetic tomography (eLORETA) cortical current density. A significant correlation between parietal alpha oscillation and somatization severity was observed in SPD patients, after accounting for the influence of anxiety and depression. Functional connectivity analysis further revealed a greater frontoparietal connectivity of the resting-state alpha oscillations in SPD patients, which was indexed by the coherence between pairs of electrodes and the linear connectivity between pairs of eLORETA cortical sources. The enhanced resting-state alpha oscillation in SPD patients could be relevant with attenuated sensory information gating and excessive integration of pain-related information, while the enhanced frontoparietal connectivity could be reflecting their sustained attention to bodily sensations and hypervigilance to somatic sensations.

## 1. Introduction

Patients with somatoform pain disorder (SPD) suffer from somatic pain (widespread pain or localized head, back, and abdominal pain) that cannot be fully explained by a specific somatic pathology [1]. Besides pain in different locations, functional complaints such as fatigue and dizziness, as well as complaints of comorbid depressive or anxiety disorders, are also common among these patients [2]. Somatosensory amplification is considered to play a crucial role in somatization [3], manifested as exaggerated and sustained attention to bodily sensations or symptoms,as well as hypervigilance to somatic sensations with excessive affect and cognitions, which intensifies these patients more distressing and alarming. Somatoform patients with greater somatization severity tend to have less behavioral activities (e.g., shopping, watching TV) requiring attention distracted from pain bodily symptoms [4, 5]. Functional neuroimaging studies revealed that patients with somatoform pain had enhanced cerebral processing of experimental nociceptive stimuli than healthy controls [6], including greater cortical activations of brain regions of pain neuromatrix (e.g., primary and secondary somatosensory cortex, amygdala, anterior insula, and inferior parietal cortex). The hyperactivations to noxious stimuli within the pain-related brain regions supported the involvement of somatosensory amplification (heightened salience to noxious percepts) in somatoform patients.

The investigation of spontaneous fluctuations of neural activities during a resting state could help identify the representation of ongoing brain state in somatoform patients. The resting-state large-scale reorganization of functional network has ever been identified among patients with SPD [7], manifested as a decreased functional connectivity between default-mode network and executive network and an increased functional connectivity between sensorimotor and frontoparietal network. Beyond the spatially extended functional network connectivity, the dynamic functional state of the brain can also be well represented by the spontaneous cortical oscillations in multiple frequency bands [8] that can be effectively measured by electroencephalography (EEG) or magnetoencephalography (MEG). It has been proposed that cortical oscillations and synchronization serve the flexible routing of information flow within the brain [9], e.g., gamma (30-100 Hz) and alpha (8-13 Hz) oscillations, respectively, relevant with feedforward and feedback information processing within the brain. While the pain experience requires the integration of sensory and contextual processes, cortical oscillations have been suggested to play a crucial role in pain processing and integration. For example, the suppression of alpha oscillation induced by the nociceptive stimulation is regarded to reflect the cortical excitability within the somatosensory system [10, 11], and the prestimulus alpha oscillation is involved in the top-down controlled gating mechanism that modulates the subsequent pain perception [12, 13]. The gamma oscillation has been considered to be particularly relevant with the encoding of pain perception, i.e., pain-induced enhancement of gamma oscillation over primary somatosensory cortex could represent the subjective perception of pain stimuli [14, 15].

To investigate the abnormalities of ongoing brain states in chronic pain patients, the analysis of cortical oscillations is also conceptually promising and methodologically well suited. While alterations of resting-state cortical oscillations have been reported for many types of chronic pain patients, the obtained results were not fully consistent. The increased resting-state alpha oscillation in patients with multiple sclerosis has been associated with attention deficit and attenuated sensory gating [16, 17], the enhanced alpha oscillation in patients with neuropathic pain has been interpreted as cognitive dysfunction [18, 19], and the positive correlation between pain duration and alpha oscillation power among patients with chronic back pain led to the speculation for the role of alpha oscillations in pain chronification [20]. In contrast, some studies also reported the decrease of alpha oscillation power among patients with chronic low back pain [21] and patients with chronic pain after spinal cord injury [22], which has been interpreted as a dysfunction of cortical inhibition and the functional comorbidity of drowsiness among these patients. The alterations of cortical oscillations in other frequency bands were also reported for chronic pain patients. Fibromyalgia patients showed general increases in theta oscillation power at the left dorsolateral prefrontal and orbitofrontal cortex, as well as increases of beta and gamma power at insular and sensorimotor cortices [23], and these alterations were suggested to be functionally relevant with their abnormalities in the cognitive and emotional modulation of pain. Among chronic low back pain patients, a positive association between ongoing pain intensity and prefrontal beta and gamma oscillations has been reported [24], indicating the relevance of chronic low back pain with the altered gamma oscillation.

While resting-state cortical oscillations might offer diagnostic and therapeutic benefits, the alterations of resting-state EEG oscillations in patients with SPD still remained unclear. For a better understanding of ongoing brain states among the patients with SPD, the present study is aimed at identifying their abnormalities of EEG oscillations during a resting state. Resting-state EEG data with eyes open were recorded from both SPD patients and healthy controls, then their spectral power density and functional connectivity were compared using both electrode-level and source-level analysis.

## 2. Materials and Methods

### 2.1. Participants

Seventeen patients (right-handed, 5 females, 35.77 ± 1.75 years, in the range of 25 and 55 years) with SPD were recruited from the Shenzhen Municipal Sixth People's Hospital (Shenzhen, China). Inclusion criteria for patients included (1) diagnosis of SPD according to the International Classification of Diseases (ICD-10) criteria; (2) over 18 and below 60 years old; (3) right-handed; (4) Han Chinese ethnics; and (5) duration of clinical pain for at least 6 months. Seventeen age- and sex-matched healthy controls (right-handed, 8 females, 33.06 ± 1.63 years, in the range of 19 and 46 years) were recruited via advertisements posted in nearby communities. The healthy controls had no chronic/ongoing pain and did not meet the ICD-10 criteria for psychiatric diagnosis. Exclusion criteria for all participants included (1) presence of pain symptoms due to severe somatic diseases; (2) presence of major psychiatric illness, including depression, anxiety, obsessive compulsive disorder, and posttraumatic stress disorder; and (3) existence of neurological diseases such as congestive heart failure, hypertension, and cerebrovascular disease. All participants gave their written informed consent after the experimental procedure had been carefully explained. The research was approved by the local research ethics committee.

### 2.2. Questionnaires

The subjective and multidimensional experience of pain in SPD patients was quantitatively measured using the Short-Form McGill Pain Questionnaire [25]. It comprises three subscales: a pain rating index (PRI) describing the qualities of pain, a 10 cm visual analogue scale (VAS) describing the intensity of averaged daily pain during the past 2 weeks, and a present pain intensity (PPI) index describing the intensity of current pain. Both SPD patients and healthy controls were instructed to fill in (1) the somatization subscale of Symptom Check List 90-Revised Version (SCL-90-R) that measures the level of somatization severity [26]; (2) Beck Depression Inventory (BDI) that measures cognitive and endogenous aspects of depression [27]; and (3) State-Trait Anxiety Inventory (STAI) that measures transitory or situational level of anxiety symptoms [28].

### 2.3. EEG Recording

Participants were seated in a comfortable chair in a silent and temperature-controlled room. They were instructed to keep eyes open and view a fixation cross in the center of a computer screen, throughout the experiment. Five minutes of continuous EEG data were recorded using 64 Ag-AgCl scalp electrodes placed according to the International 10-20 system (Brain Products GmbH; Munich, Germany; band pass: 0.01–100 Hz; sampling rate: 1000 Hz), using the FCz as recording reference. Electrode impedances were kept below 10 k*Ω*. Electrooculographic (EOG) signals were simultaneously recorded using two surface electrodes to monitor ocular movements and eyeblinks, one placed ~10 mm below the left eye and the other placed ~10 mm from the outer canthus of the left eye.

### 2.4. EEG Data Preprocessing

EEG data were preprocessed using EEGLAB, an open source toolbox running under the MATLAB environment (The MathWorks Inc., Natick, Massachusetts, United States). Electrodes located on the neck/face were removed, and the bad electrodes with huge jumps or completely flat were interpolated using a spherical spline method. Continuous EEG data were band-pass filtered (1-45 Hz) using a Hamming finite impulse response filter (filter order: 3300; transition band width: 1 Hz; cutoff frequencies: 0.5-45.5 Hz). Then, the continuous EEG data were segmented into consecutive 2-second epochs, and epochs with the amplitude values exceeding ±80 *μ*V at any electrode were further rejected. The resulting number of EEG epochs was not different between patients and controls (130.12 ± 1.89 s vs. 127.15 ± 1.15 s, *p* = 0.20, *t* = 1.29). Epochs contaminated by eyeblinks and movements were corrected using an independent component analysis algorithm [28]. In all datasets, independent components with a large electrooculogram (EOG) contribution and a frontal scalp distribution were removed [28]. Finally, the artifact-free resting-state EEG were rereferenced to the common average for further processing, to harmonize the resting-state EEG data collected with different reference electrodes and make our results more comparable with other studies [16, 22, 29, 30].

### 2.5. Estimation of Resting-State EEG Oscillations

Preprocessed EEG signals were transformed to the frequency domain using a Fast Fourier Transform (FFT, Welch algorithm, Hanning window, no phase shift, 0.5 Hz frequency resolution), yielding an EEG spectrum ranging from 1 to 45 Hz. For each participant, the spectra of each electrode were averaged across epochs. To identify the frequency intervals within which spectral power showed significantly different between healthy controls and SPD patients, we adopted point-by-point statistical analyses (e.g., for each frequency point) combined with nonparametric permutation approach [31]. The spectral power density, measured at parietal electrodes (Pz, P1, P2, P3, P4, P5, and P6) exhibiting the most prominent oscillation during wakeful rest [32, 33], was compared between healthy controls and SPD patients on each frequency point, thus yielding a map of *t* values for each frequency point. To account for the multiple comparison problem in the point-by-point analysis, a cluster-level nonparametrical permutation testing was performed [31]. Specifically, significant frequency points (*p* < 0.05) were categorized in clusters based on their frequency adjacency (cluster-level statistical analysis). Only the cluster with the largest number of significant frequency points was selected to control for false-positive observations. We performed 5000 random permutations, thus generating the cluster-level permutation *t* statistics. The cluster-level statistics were defined by calculating the sum of the *t* values of all frequency points within the cluster, and the two-tailed *p* values were derived by locating the observed cluster-level *t* statistics under the estimated permutation distribution. In order to confirm the identified frequency interval, a region of interest- (ROI-) based statistical analyses were further performed. Within this frequency interval, the average spectral power density within the frontal, central, parietal, occipital, and temporal electrode ROIs (the electrodes for each ROI are summarized in Table 1) was computed for each participant. Electrode-level spectral power density within the alpha frequency band was compared between patients and controls, using mixed-design repeated measures analysis of variance (ANOVA) with factors of “group” (patients and controls) and “ROI” (frontal, central, parietal, occipital, and temporal ROIs).

To further localize the cortical current density of the atypical EEG oscillations in patients, we used the freeware called exact low-resolution brain electromagnetic tomography, eLORETA [34], which represents the improved version of the previous pieces of software called LORETA [35] and standardized LORETA [36]. It used a head volume conductor model composed of the scalp, skull, and brain and solved the so-called EEG inverse problem estimating “neural” current density values at any cortical voxel of the head volume conductor model. The brain model was based on a realistic cerebral shape taken from a template of the Montreal Neurological Institute average MRI brain map (MNI152) [37]. The input for source estimation was the EEG spectral power density computed at scalp electrode level, and the output was the electrical brain source space formed by 6239 voxels at 5 × 5 × 5 mm spatial resolution, restricted to the cortical grey matter. The current source density of the eLORETA cortical functioning image was calculated for each patient and healthy control, within the identified frequency interval. Further, eLORETA cortical current source density between patients and controls was compared in different brain regions. Consistent with electrode-level ROIs, we defined source-level ROIs including the frontal, central, parietal, occipital, and temporal regions. The respective Brodmann areas of each source-level ROI are summarized in Table 1. Source-level eLORETA cortical current density of resting-state alpha oscillations was compared between patients and controls, using mixed-design repeated measures ANOVA with factors of “group” (patients and controls) and “ROI” (frontal, central, parietal, occipital, and temporal ROIs).

### 2.6. Estimation of Resting-State EEG Functional Connectivity

The functional connectivity of the identified brain region showing significant between-group difference of cortical oscillations was further investigated on both electrode- and source-level. Specifically, the electrode-level functional connectivity of the averaged alpha oscillation in the parietal ROI with other electrode ROIs was assessed using coherence which estimates the linear time-invariant relationship between time series [38]. The coherence was computed as the squared cross-spectrum of two time series, divided by the power spectra of both time series [38], yielding a value between 0 (indicating no linear relationship) and 1 (indicating perfect linear relationship). The source-level functional connectivity between pairs of cortical ROIs was estimated using lagged linear connectivity (LLC) as implemented in the eLORETA statistical package [39]. The LLC estimation provides linear measurements of statistical interdependence between pairs of eLORETA cortical activations [39]. Notably, the LLC estimation depicts the connectivity of two signals after excluding zero-lag instantaneous artifacts that are not often associated with the true physiological interactions, and has been showed to be resistant to nonphysiological artifacts' particularly low spatial resolution and volume conduction [40, 41]. The resulting functional connectivity measures (both coherence and LLC) between the parietal and other brain regions were converted to *z* values using the Fisher's *r*-to-*z* transformation and were separately compared using mixed-design repeated measures ANOVA with factors of “group” (patients and controls) and “ROI” (frontal, central, occipital, and temporal ROIs).

## 3. Results

### 3.1. Psychometric Results

As summarized in Table 2, SPD patients were suffering from moderate pain and had significantly greater somatization (*p* < 0.001), state-anxiety (*p* < 0.001), trait-anxiety (*p* < 0.001), and depression scores (*p* < 0.001). Correlation analysis across all SPD patients revealed that the pain severity was significantly and positively correlated with somatization (*r* = 0.66, *p* = 0.004) and depression (*r* = 0.64, *p* = 0.006). It indicates that SPD patients suffering from greater pain tend to have greater somatization severity and depression symptoms.

### 3.2. Resting-State EEG Oscillations

**Figure 1:**
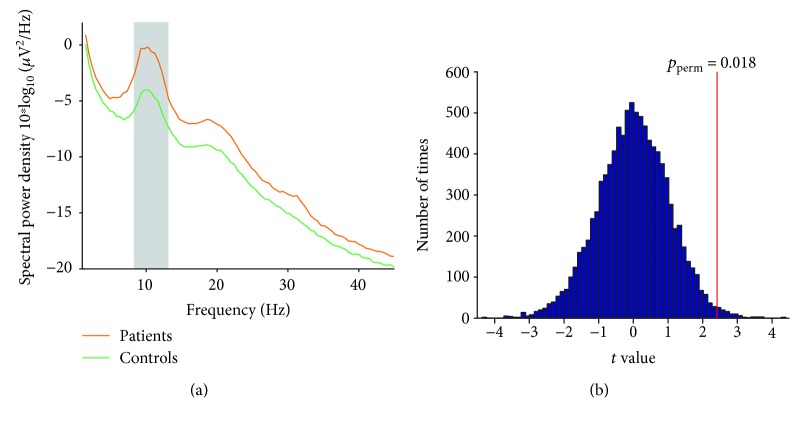
Comparisons of resting-state EEG oscillations for patients and controls. Grand average resting-state spectral power density (measured at parietal electrodes (a)) in the frequency range of 1-45 Hz for SPD patients (orange line) and healthy controls (green line). Within the frequency interval 8.5-12.5 Hz (marked using a grey rectangle), patients exhibited significantly greater spectral power density than healthy controls (*t* value, vertical red line (b); *p* = 0.018; 5,000 permutations).

**Figure 2:**
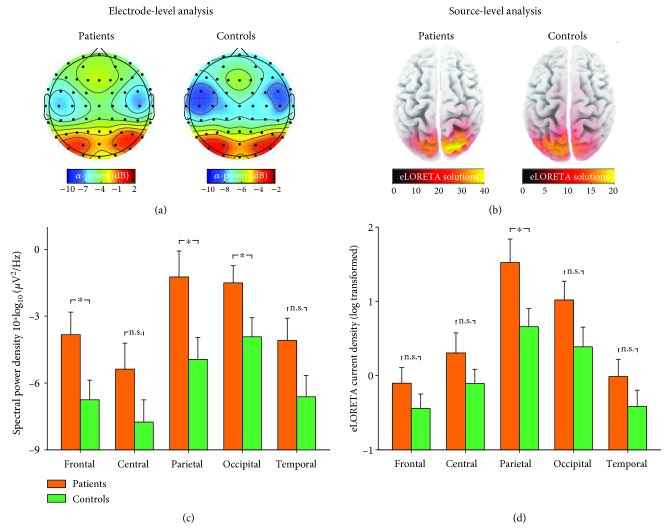
Resting-state EEG alpha oscillations for patients and controls. Group averages of electrode-level spectral power density (a, c) and eLORETA cortical current density (b, d) for resting-state EEG alpha oscillations. The scalp topographies of resting-state alpha oscillations were maximal at the parietal region for both SPD patients and healthy controls, as confirmed by electrode-level and source-level spatial distributions. For a displaying purpose, the color map limits were adjusted for each group. Data are expressed using Mean ± SEM. ^∗^*p* < 0.05; n.s.: *p* > 0.05, independent-sample *t*-test.

Point-by-point statistical analysis combined with nonparametrical permutation testing (5,000 times) indicated that patients had significantly greater resting-state EEG spectral power density within the 8.5-12.5 Hz (i.e., alpha) frequency interval (*p*_perm_ = 0.018, Figure 1). The topographies of spectral power density for alpha oscillations were maximal over parietal electrodes for both patients and controls (Figure 2(a)). The average values of alpha power density at different regions were summarized in Figure 2(c). The ANOVA revealed a main effect of “group” (*F* = 4.29, *p* = 0.047, *η*_p_^2^ = 0.12) where patients showed greater alpha oscillatory power and a main effect of “ROI” (*F* = 61.31, *p* < 0.001, *η*_p_^2^ = 0.66) where alpha oscillatory power at parietal and occipital electrodes was greater. The interaction between factors of “group” and “ROI” was not significant (*F* = 1.80, *p* = 0.17, *η*_p_^2^ = 0.055). Independent-sample *t*-test revealed that the EEG alpha oscillations in patients were in greater power than healthy controls at frontal, parietal, and occipital electrodes (*p* = 0.036, *p* = 0.022, and *p* = 0.044, respectively, Figure 2(c)) but not significantly different at central and temporal electrodes (*p* = 0.13 and *p* = 0.077, respectively).

The source-level eLORETA cortical current density for resting-state alpha oscillation also showed maximal distribution over the parietal lobe for both patients and controls (Figure 2(b)). The average values of eLORETA current density (log transformed) at different cortical regions were summarized in Figure 2(d) and were compared using ANOVA with factors of “ROI” and “group.” It revealed a main effect of “ROI” (*F* = 50.31, *p* < 0.001, *η*_p_^2^ = 0.62) where alpha oscillatory power at the parietal and occipital lobes were greater, but the main effect of “group” was marginally significant (*F* = 2.93, *p* = 0.097, *η*_p_^2^ = 0.086) suggesting that patients tend to exhibit greater alpha oscillation power. The interaction between factors of “group” and “ROI” was not significant (*F* = 1.67, *p* = 0.19, *η*_p_^2^ = 0.051). Independent-sample *t*-test revealed that the eLORETA current source density of patients was significantly greater at the parietal lobe (*p* = 0.039, Figure 2(d)), but not at the frontal, central, occipital, and temporal lobes (*p* = 0.24, *p* = 0.23, *p* = 0.09, and *p* = 0.22, respectively), as compared to controls. This result further confirmed the atypical resting-state alpha oscillations at the parietal region in patients, which has been identified by electrode-level spectral analysis.

**Figure 3:**
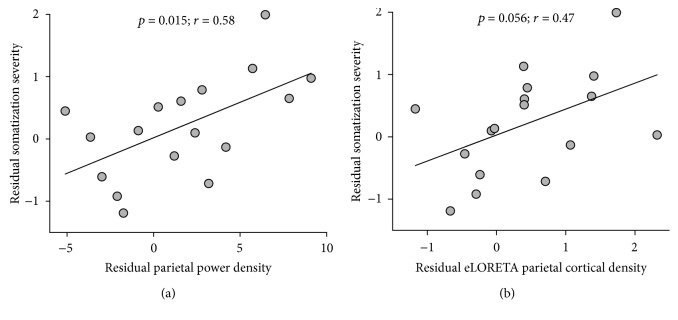
Relationship between somatization severity and resting-state alpha oscillation in SPD patients. After accounting for the influence of anxiety and depression, the residual somatization severity was significantly correlated with residual alpha oscillation power density at parietal electrodes and marginally significantly correlated with the residual eLORETA cortical current density at the parietal lobe. Each grey dot represents values from a single SPD patient, and black lines represent the best linear fit.

After accounting for the influence of anxiety and depression on somatization severity and resting-state alpha oscillation, the residual somatization severity and parietal alpha oscillation (electrode-level spectral power density and eLORETA cortical current density) were obtained using linear regression analysis. Across all the patients, the residual somatization severity was significantly and positively correlated with the alpha oscillation power density at parietal electrodes (*r* = 0.58, *p* = 0.015, Figure 3) and marginally correlated with the eLORETA cortical current density at the parietal lobe (*r* = 0.47, *p* = 0.056, Figure 3). It suggests that SPD patients with greater somatization severity tend to have a greater resting-state alpha oscillation.

### 3.3. Resting-State EEG Functional Connectivity

**Figure 4:**
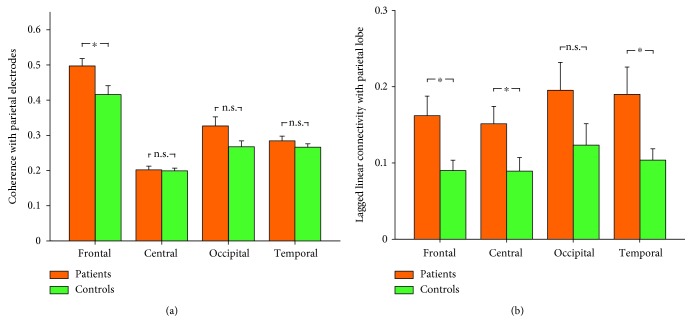
Functional connectivity with the parietal region for resting-state alpha oscillations between patients and controls. The functional connectivity for resting-state alpha oscillatory activity was assessed using coherence between pairs of electrodes (a) and using lagged linear connectivity between pairs of cortical regions (b). Data are expressed using Mean ± SEM. ^∗^*p* < 0.05; n.s.: *p* > 0.05, independent-sample *t*-test.

We further assessed the functional connectivity of the parietal region using coherence between pairs of electrode ROIs and LLC between pairs of cortical ROIs, within the alpha frequency band. Mixed-design ANOVA on coherence measures revealed a significant interaction between factors of “group” and “ROI” (*F* = 4.25, *p* = 0.018, *η*_p_^2^ = 0.12). Post hoc independent-sample *t*-test showed that SPD patients had greater functional connectivity between the parietal and frontal regions (*p* = 0.018) than healthy controls, while the functional connectivity of the parietal region with the central, temporal, and occipital regions was not different between patients and controls (*p* > 0.05 for all comparisons, Figure 4(a)). Mixed-design ANOVA on LLC measures revealed a significant main effect of “group” (*F* = 6.52, *p* = 0.016, *η*_p_^2^ = 0.17) indicating that patients had greater functional connectivity than healthy controls. As summarized Figure 4(b), compared with healthy controls, the parietal functional connectivity was greater with the frontal (*p* = 0.021), central (*p* = 0.042), and temporal lobes (*p* = 0.038) for patients with SPD, but not with the occipital lobe (*p* = 0.13).

## 4. Discussion

The present study recorded EEG data from SPD patients and healthy controls during a resting state with eyes open, and their cortical oscillations, as well as functional connectivity, were compared based on electrode-level and source-level analysis. We have obtained two main findings. First, compared with controls, patients with SPD exhibited greater alpha oscillations (8.5-12.5 Hz) at the parietal region, as confirmed by both electrode-level spectral power density and source-level eLORETA cortical current density. Second, patients showed greater frontoparietal connectivity for the resting-state alpha oscillatory signals, as confirmed by the coherence between pairs of electrodes and the LLC between pairs of eLORETA cortical sources. These alterations demonstrated the frequency-dependent hyperactivation of resting-state EEG oscillations among the SPD patients, which could implicate the hypersensitive bodily attention and somatosensory amplification in these patients. This understanding might offer diagnostic and therapeutic benefits for the sustained somatoform pain.

The experience of pain has been associated with cortical oscillations in different frequency bands that could represent the dynamic interactions between sensory and contextual processes [9]. Alterations of cortical oscillations have been linked with the pathological abnormalities of chronic pain patients [42], but the results are not fully consistent. The most-noticed abnormality is the increase of theta oscillation in chronic pain patients such as neurogenic pain [18, 43], which has been proposed to be relevant with thalamocortical dysrhythmia [44]. However, we also did not observe abnormalities in theta oscillations in patients with somatoform pain, which is similar with results from chronic pain patients with spinal cord injury [22] or chronic back pain [20]. This inconsistence could be arising from the different types of chronic pain patients involved in these clinical studies and the different EEG recording paradigms (eyes-close or eyes-open state with different durations), as well as the different pain comorbidities such as anxiety, depression, and sleeping disorders which could also influence the resting-state cortical oscillations [45–47].

An increase of the oscillation at alpha band frequency has ever been reported in chronic pain patients [17, 43]. For example, among patients with multiple sclerosis, Kim et al. observed an increase of alpha oscillation power within several nodes of the salience network and ascending nociceptive pathway [17], which was interpreted as a result of overflowing sensory information due to a reduced sensory gating. The increased alpha oscillation has also been identified among migraine patients, suggesting an overintegration of sensory information in these patients [48]. The increase of alpha oscillation in chronic pain patients is in line with the findings from animal models of acute, inflammatory, and neuropathic pain, which exhibited increased cortical oscillation within broad frequency band (3-30 Hz) over the primary somatosensory cortex and the prefrontal cortex [49, 50]. Well consistent with these results, SPD patients had increased alpha oscillations with maximal distribution over the parietal region, as confirmed by both electrode-level spectral power density and source-level eLORETA cortical current density. Considering the functional role of alpha oscillations in local inhibition and sensory gating [17, 51, 52], the increased alpha oscillations in SPD patients could be reflecting the attenuated sensory information gating and excessive integration of pain-related information, thus appeared as somatosensory amplification in these patients. This hypothesis was further supported by the positive correlation between resting-state alpha oscillation power and somatization severity across the SPD patients, where somatization severity and somatosensory amplification are closely linked [53, 54]. Although the observed increased alpha oscillation among SPD patients was consistent with findings from many chronic pain studies [17, 19, 43, 55], it was in contrast with experimental pain studies [12, 13, 56] showing the negative correlation between prestimulus alpha oscillation and subsequent pain perception. This could be arising from the differences between experimental pain (well controlled with limited duration and specified location) and clinical pain (long lasting and widespread, with pain interference such as sleep interference), particularly considering the absence of organic pathology involved in the maintenance of chronic pain suffered by SPD patients.

After identifying the enhanced alpha oscillations at the parietal region among the SPD patients, we further investigated the parietal functional connectivity for the resting-state alpha oscillatory signals. Compared with healthy controls, patients with SPD also exhibited greater frontoparietal connectivity, as confirmed by (1) coherence for the alpha oscillatory signals measured at parietal and frontal electrodes and (2) LLC estimations for the alpha oscillatory eLORETA solutions estimated at the parietal and frontal lobes. Human frontoparietal functional connectivity has long been implicated in allocating attention resources to perceptual or internal representations, such that attention can adaptively be directed towards external stimuli as well as internal representations [57]. The frontoparietal functional connectivity showed to be correlated with expectancy-induced pain modulation [58], thus suggesting the association between frontoparietal connectivity and endogenous pain modulation. Combining the understanding that somatoform patients typically exhibit excessive attention to their bodily sensations and intensify them as more alerting and distressing [53, 59], the observed enhanced frontoparietal connectivity might represent the sustained bodily attention, hypervigilance to somatic sensations, and/or the dysfunction of pain modulation systems among the SPD patients. Indeed, our result was in line with the enhanced medial prefrontal cortex functional connectivity with the posterior cingulate cortex and precuneus among patients with idiopathic temporomandibular disorder, which was linked with pain rumination across the chronic pain patients [60].

Several limitations should be noted in the present study. First, while EEG data were recorded from the patients and controls during a resting state with eyes open, we could not completely rule out the possibility that the identified abnormalities of resting-state cortical oscillations were relevant with their drowsiness or eyes closing. Second, the sample size of patients is limited, and the relationship between somatoform pain and resting-state cortical oscillations should be further investigated based on a large sample of patients. Third, while we have identified the alterations of resting-state alpha oscillations among SPD patients, the exact role of the altered alpha oscillation in the maintenance of somatoform pain is still unclear. This deserves to be further investigated using simultaneous electrophysiological recordings combined with pharmacological interventions.

In summary, this study provides evidence for enhanced resting-state alpha oscillations and frontoparietal connectivity among patients with SPD, which could be functionally relevant with the sustained attention to bodily sensations and hypervigilance to somatic sensations in these patients. Further studies are needed to validate these findings in a larger cohort of patients and to identify the exact role of the atypical oscillations in the maintenance of somatoform pain.

## Figures and Tables

**Table 1:** Definitions of electrode-level and source-level regions of interests (ROIs).

	Electrode-level (electrodes)	Source-level (Brodmann areas)
Frontal	Fz, F1, F2, F3, F4, F5, F6, F7, F8	8, 9, 10, 11, 44, 45, 46, 47
Central	Cz, C1, C2, C3, C4, C5, C6	1, 2, 3, 4, 6
Parietal	Pz, P1, P2, P3, P4, P5, P6	5, 7, 30 39, 40 43
Occipital	Oz, O1, O2	17, 18, 19
Temporal	T7, T8, FT7, FT8, TP7, TP8	20 21, 22, 37, 38, 41, 42

**Table 2:** Psychometric variables for patients with SPD and healthy controls.

	Patients	Controls	Statistics
Pain rating index (PRI)	10.82 ± 1.49	—	—
Visual analogue scale (VAS)	47.65 ± 4.37	—	—
Present pain intensity (PPI)	1.71 ± 0.28	—	—
Somatization score (SCL-90-R)	1.62 ± 0.21	0.51 ± 0.10	*p* < 0.001, *t* = 4.70
Depression (BDI)	8.18 ± 1.07	2.88 ± 0.84	*p* < 0.001, *t* = 3.89
State-anxiety (STAI-S)	42.88 ± 2.51	31.71 ± 2.13	*p* < 0.001, *t* = 3.40
Trait-anxiety (STAI-T)	52.24 ± 2.69	34.53 ± 2.60	*p* < 0.001, *t* = 4.74

## Data Availability

The datasets used to support the findings of this study are available from the corresponding author on reasonable request.
